# Electric Fields at the Lipid Membrane Interface

**DOI:** 10.3390/membranes13110883

**Published:** 2023-11-16

**Authors:** Yury A. Ermakov

**Affiliations:** Frumkin Institute of Physical Chemistry and Electrochemistry, Russian Academy of Sciences, Moscow 119071, Russia; yury.a.ermakov@gmail.com

**Keywords:** electrical double layer, liposomes, planar bilayer lipid membranes, lipid monolayers, zeta and Volta potentials, boundary, surface and dipole potentials, electrokinetic measurements, intramembranous field compensation, adsorption of ions and membrane active substances

## Abstract

This review presents a comprehensive analysis of electric field distribution at the water–lipid membrane interface in the context of its relationship to various biochemical problems. The main attention is paid to the methodological aspects of bioelectrochemical techniques and quantitative analysis of electrical phenomena caused by the ionization and hydration of the membrane–water interface associated with the phase state of lipids. One of the objectives is to show the unique possibility of controlling changes in the structure of the lipid bilayer initiated by various membrane-active agents that results in electrostatic phenomena at the surface of lipid models of biomembranes—liposomes, planar lipid bilayer membranes (BLMs) and monolayers. A set of complicated experimental facts revealed in different years is analyzed here in order of increasing complexity: from the adsorption of biologically significant inorganic ions and phase rearrangements in the presence of multivalent cations to the adsorption and incorporation of pharmacologically significant compounds into the lipid bilayer, and formation of the layers of macromolecules of different types.

## 1. Introduction

Lipid models of cell membranes have become a convenient object for studying electric field distribution at the interface of biological membranes. This generally consists of two parts of a different molecular nature. One is a potential drop in the diffuse part of the electrical double layer (EDL) between what is called the external Helmholtz plane and the bulk of the electrolyte. It is naturally to name this the surface potential, *φ_s_*. Another part is commonly associated with a difference between the internal and external Helmholtz planes and results from immersion of some ions and charged molecules into the membrane, affecting the orientation of dipole moments in the lipid polar heads and the water molecules associated with them. This part is named the dipole potential, *φ_d_*. The sum of *φ_s_* and *φ_d_* gives us the boundary potential, *φ_b_*. Its absolute value is not available for direct measurement but its variations are suitable for detection. That is why an important task delegated to bioelectrochemical researchers is to associate any changes in the boundary potential with both of its components and to prove their relationship with the structure of the membrane–water interface. This task underlies the study of the objects proposed below, selected on the basis of their significance for certain biomedical problems. The knowledge gained by author and colleagues at different stages of this study is published for a several decades and summarized in previous reviews [1,2].

Electric fields at membrane interfaces are of particular interest for many biological applications. The surface of cell membranes is the region of many biochemical and transport processes, where ions and charged molecules work as some active agents. The literature contains a huge number of original studies, reviews, and monographs devoted to various physicochemical features of lipid models of cell membranes. Theoretical and experimental studies of electrostatic phenomena at the surface of lipid membranes and their environment are described in sufficient detail in [3,4,5,6,7]. For instance, the excellent monograph by G. Cevc [3] contains information about some fundamental theoretical models that describe the charging and hydration of phospholipid membranes in the presence of inorganic ions, as well as the polarization of the aqueous environment by a potential drop in the immediate vicinity of the membrane. These factors significantly affect the interaction of membranes as they approach each other and the appearance of so-called “hydration forces”, which manifest themselves in these interactions [4]. In this review, problems of membrane interactions are left to one side and the focus is limited to the consideration of experimental data that indicates the effect of membrane-active ions and substances adsorbed at the surface of lipid membranes on the distribution of electric fields, primarily directly in their polar region. These phenomena have been actively studied by many authors who have developed an extensive arsenal of experimental approaches to assess the physicochemical parameters of lipid membranes. Some of them have a significant impact on the development of the bioelectrochemical methods described below [5,6,7,8,9,10,11,12,13,14,15]. Judging by recent publications (see, for example, [16]), the structure of lipid membranes and their outer boundary is still in the focus of many researchers, who apply their efforts to create new techniques and to improve the theoretical analysis of electrostatic phenomena. Biologically important substances with their membrane activity, ionized proteins, and polypeptides would require much more space for detailed analysis than a short review can afford. Therefore, the current review illustrates the data for some biologically significant ions and molecules that have been revealed by bioelectrochemical methods, mainly developed with the participation of the author, and which may be of particular interest for solving some of the problems of cell membrane biochemistry.

## 2. Electric Fields at the Interfaces of Lipid Membranes

The lipid component of cell membranes consists mainly of phospholipids, and model membranes suitable for simulating the bilayer structure of the lipid matrix of cellular ones are liposomes and planar bilayer lipid membranes (BLM), while the technique of lipid monolayers enables the study of lipid packing. Modern ideas about the distribution of the electric field at the boundaries of such membranes take into account the difference in the dielectric properties of hydrophobic and hydrophilic regions, which correlate with the molecular structure of the membranes in the direction normal to its surface. Electrochemical methods make it possible to measure the average values of the electric potential in planes, the position and physical meaning of which depend on the method used and the device setup. One of the fundamental sources of information about the ionization of polar groups at the surface of membranes and the magnitude of the surface charge are electrokinetic measurements. However, the limited accuracy of real experiments does not allow us to describe the results by theoretical models that include a large number of parameters. That is why the most effective quantitative analysis of electrokinetic data is accessible by the classical model of the EDL proposed at the beginning of the last century by M. Gouy [17] and D.L. Chapman [18]. Later it was modified in O. Stern’s work [19] with a description of the ion adsorption by Langmuir relations. D.C. Grahame developed a significant contribution to the theory of EDL and the generalization of equations for electrolytes of arbitrary composition [20]. In subsequent years, there have been many attempts to construct more complex physical models of the structural organization of the interface boundaries (see, for example, [21]). Naturally, there are many physical parameters suitable for describing the molecular structure of membranes and their interface. This is especially important for comparing different types of membrane models such as liposomes, BLMs, and monolayers. On the other hand, the accuracy of experimental data is very limited and allows the choice of a small number of free parameters to approximate the data. Fortunately, this is sufficient for the vast majority of cases, and the simplest version of the classical Gouy–Chapman–Stern (GCS) model describes well all experimental results for various lipid models of biomembranes [22]. This circumstance is illustrated below through various examples.

**Figure 1:**
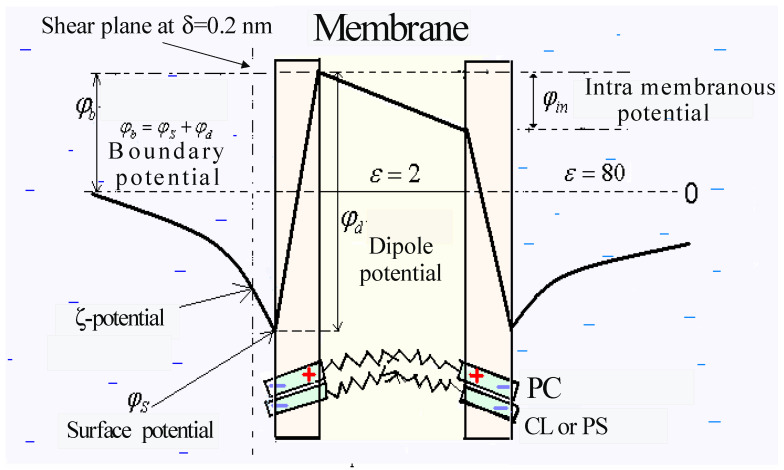
Distribution of electric potentials in the membrane and at its boundaries with aqueous electrolytes.

The potential drop inside the polar region of the membrane can be described as the potential difference between internal and external Helmholtz planes at the membrane interface (Figure 1). It depends on the location of hydrophilic groups of phospholipids, on the orientation of their dipole moments, on the amount and distribution of associated water molecules, on the position of the adsorption plane for hydrophobic ions and ionized molecules, etc. This part of the boundary potential is mainly of a dipole nature and its absolute value is not available for direct measurement. Fortunately, the presence of the dipole component of the boundary potential can be judged by comparing the data of electrokinetic measurements with alternative methods that detect the total jump in the electric potential at the boundary of the membrane that we defined as the boundary potential.

Generally, the total electric potential difference between the volume of the aqueous phase and the inner hydrophobic region of the membrane (boundary potential) can be presented as the sum of the surface and dipole potentials: *φ_b_* = *φ_s_* + *φ_d_*, measured as a potential jump between two planes (Figure 1). The first plane is conditionally assigned to the center of the hydrophobic region of the lipid bilayer or is located in the air outside the region of hydrocarbon chains when lipids cover the aqueous subphase by a monolayer. In both cases, the second plane is located in the aqueous solution at a considerable distance from the interface, where the number of cations and anions is equal, and the electric potential is assumed to be zero. The potential jump between the external surface of the polar region of lipids and the aqueous solution, *φ_s_*, is hereinafter attributed to the diffuse part of the electrical double layer and is called the surface potential. The electric field inside the region of lipid polar headgroups usually depends on the possible immersion of ions to some depth into the lipid layer and the state of hydration of the polar groups. Thus, the potential drop in this region is named the boundary potential, *φ_d_*. It is this component that is most closely related to the structure of the interfacial boundary, and therefore its subtraction from the total boundary potential is of particular interest.

## 3. Electrokinetic Measurements in Liposome Suspension

The surface potential is available for direct measurement by electrokinetic methods. These are described in detail in [23]. With regard to phospholipid liposomes, the simplest measurement procedure and processing of results within the framework of the GCS model has been developed by S. McLaughlin in his works [12,22]. In particular, in [24], a series of measurements convincingly prove the applicability of a quantitative description of the results in the framework of this relatively simple model. Based on the objects chosen for measurement and the analysis protocol, results of that study may serve as the best test for verification of any methods developed to acquire the electrokinetic data of colloidal suspensions and particularly by the dynamic light scattering (DLS) technique. Despite the fact that this method and the measurement procedure are complicated, and the evaluation of results is not obvious, the experimental data show good agreement with traditional methods [25]. Thus, this test may serve to confirm the efficiency of any new approaches for the measurement of electrokinetic phenomena. This is important because some data obtained with similar equipment may differ noticeably from those published earlier. It may lead some authors to erroneous conclusions about the nature of liposome surface ionization, which differs significantly from classical ideas about the mechanism of EDL formation (see [26]).

**Figure 2:**
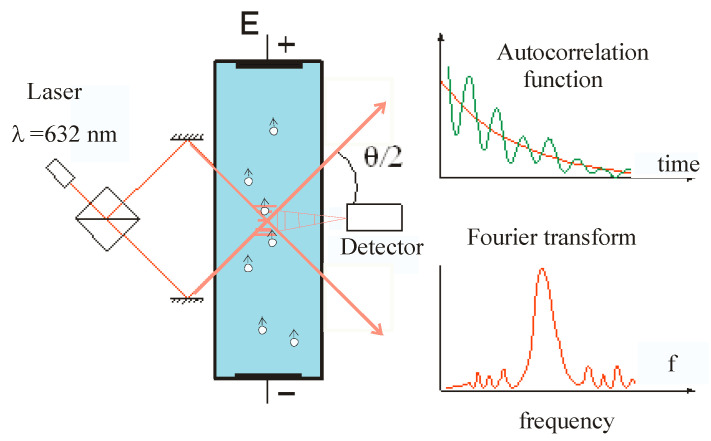
Schematic representation of the procedure of electrokinetic measurements using registration of dynamic light scattering by the ELS method.

The physical principle of electrokinetic measurements via dynamic light scattering in a colloidal suspension is based on numerical analysis of the shape of correlation curves. There are two possibilities to obtain these curves: registration of autocorrelation amplitude dependences (electrokinetic light scattering, the ELS method), which is typical for early devices, and by phase shift detection between the reference and measuring laser beams (phase analysis of light scattering, the PALS method), used in modern equipment from different companies. To understand the general principle of measurements and their quantitative processing, it is convenient to use the capabilities of the ELS technique. To control the directed of motion of particles in the applied electric field, which is based on the autocorrelation function of light pulses scattered at some point in the depth of the measuring cell. Two laser beams intersect at the angle *θ* and a system of interference fringes is formed, which depends on the wavelength of light, *λ* (Figure 2). When colloidal particles move across these bands in the external electric field E, periodic fluctuations in the electrical signal with a frequency, *f*, are recorded by a detector, and are directly related to the velocity of the directed movement of particles along the field. This is proportional to the electrophoretic mobility of the colloidal particle, *μ*. To estimate the electric potential, a special value called *ζ*-potential, is introduced, taking into account the hydrodynamic conditions, dielectric properties, *εε_0_*, and viscosity, *η*, of the aqueous media (1). In most conditions related to biochemical systems, particle sizes far exceed the length of the Debye screening and this potential can be evaluated according to the Smoluchowski’s theory (see [23] for details). However, it should be understood that, in the general case, its value does not coincide with the potential drop in the diffuse part of the EDL because of the unknown position of the hydrodynamic shear plane located close to the surface.

(1) $$f\frac{\lambda }{2Esin\frac{\theta }{2}}=\mu =\zeta \frac{\varepsilon \varepsilon_{0}}{\eta }$$

Signal fluctuations are reflected in the shape of the autocorrelation function, which is numerically processed by Fourier transform, and the mobility spectrum of colloidal particles is determined by the frequency scale proportional to the *ζ*-potential of the particles. It is important to note that the point of intersection of laser beams should be moved to a certain distance from the cell wall, to be located at a stationary plane where, according to the hydrodynamic conditions, the liquid remains stationary and, thereby, the effect of electrodiffusion is eliminated. Unfortunately, the small dimensions of the cell do not allow us to fix this position, which in some devices requires a special measurement procedure for an approximate assessment of the contribution of electrodiffusion. In particular, this circumstance may affect the determination of the point of zero charge of the colloidal particle.

In most cases, the suspension of multilamellar liposomes is prepared by drying the lipid solution under vacuum in a round bottom flask, and then by vigorous shaking in the aqueous solution. The suspension is not monodisperse and the average size of multilayer liposomes appears to be in the range of 0.5–1.0 μm. Colloidal particles of the size comparable with the Debye screening length require a rather complicated analysis of the electrokinetic data [23]. However, in biochemical environments, the ionic strength of the electrolyte is usually much higher than 1 mM. Therefore, the Debye screening length does not exceed 10 nm, and the electric double layer near the surface of liposomes can be considered flat, as is suggested by Smoluchowski’s theory. With a sufficiently high ionic strength of electrolyte, one needs to take into account the position of the hydrodynamic shear plane, *δ*, at some distance from the surface that requires an independent definition. In the works of S. McLaughlin, this distance is assumed to be 0.2 nm, which makes it possible to obtain good agreement between the electrokinetic data and the predictions of the GCS model for both monovalent [24] and divalent [27] electrolyte solutions. Moreover, this approach has been the subject of detailed analysis in subsequent works by the author of the special review that proved its applicability to many biologically important objects [28]. A similar conclusion about the adjustable value of *δ* also follows from a comparison of the electrokinetic data with the boundary potential of planar BLM detected by the method of intramembranouse field compensation (IFC), described below.

It is well known that the electrical charge of biomembranes is predominantly determined by their lipid composition and the presence of anionic phospholipids, of which the most common is phosphatidylserine, PS, which is involved in the formation of intracellular and intercellular signal contacts [29] and is one of the first signs of apoptosis [30]. From the methodological point of view, one can be convinced that the change in the surface charge of the membranes containing PS and uncharged lipids like phosphatidylcholine, PC, is proportional to the amount of PS in the mixture. This lipid composition is suggested in [24] and used in [25] for experimental verification of the equipment. Thus, the relationship of the surface potential *φ*(0) with the surface charge density *σ* and the concentration of the symmetric electrolyte *c_bulk_*, in which anions and cations have the same charge *z*, is given by the expression:

(2) $$\sigma_{0}=\sqrt{8kT\varepsilon \varepsilon_{0}c_{bulk}}sinh\left(\frac{ez\varphi \left(0\right)}{2kT}\right),$$

and the potential distribution *φ* (*x*) near the charged surface is described by the relation:

(3) $$tanh\left(\frac{ez\varphi \left(x\right)}{4kT}\right)=e^{-kx}tanh\left(\frac{ez\varphi \left(0\right)}{4kT}\right),$$

which takes into account the Debye screening length, 1/*k* in solution, and the electron charge *e*

(4) $$k=\sqrt{\frac{2z^{2}e^{2}c}{\varepsilon \varepsilon_{0}kT}}.$$

The case of asymmetrical electrolyte is given in [9].

An adsorption of cations on negatively charged phospholipids (e.g., PS) is described by a simple Langmuir isotherm. If one takes into account that anionic lipids are ionized at a low content of hydrogen ions (normal pH), then for an adequate calculation of the surface potential and charge, the isotherm takes the form:

(5) $$\frac{\sigma }{\sigma_{max}}=\frac{1}{1+K_{1}c_{1}\left(0\right)+K_{2}c_{2}\left(0\right)},$$

where the product *K_i_c_i_*(0) reflects the fraction of the surface occupied by monovalent cations of the supporting electrolyte and protons that fills binding sites associated with a single charge of the phospholipid (here *K_i_* is the equilibrium constant of cation adsorption, and *c_i_*(0) is their concentration at the surface). The value of surface charge density *σ_max_* corresponds to the maximal density of anionic phospholipids. This value was fixed in [24] for any types of cations to be equal to 0.23 C/m^2^, which corresponds to an area of 70 Å^2^ occupied by one lipid molecule at the bilayer surface. In our calculations in [25], this value, as well as the binding constants, have been treated as individual parameters for each type of cation. This type of experimental curve approximation gives these values of equilibrium binding constants close to those published in [24], while the maximum charge density of free PS molecules turned out to be lower (0.15 C/m^2^) but the same for potassium and sodium cations. The value of 0.23 C/m^2^ has been found only for tetramethylammonium chloride (TMA) and choline cations. This fact reflects the different ability of organic and inorganic ions to modify the state of polar groups hydration of the phospholipid, possibly due to the partial immersion of the inorganic cations into the polar region at the individual position of the Helmholtz inner layer. This hypothesis agrees well with the results of molecular dynamics simulations [31]. The ionic equilibrium at various pH values of the electrolyte also significantly depends on the equilibrium of protons near the surface. This binding constant of hydrogen ions for PS membranes is estimated from electrokinetic data [32] to be *pK* = 3.0, which is several orders of magnitude lower than that of potassium cations. Therefore, in most calculations, the equilibrium of hydrogen near the surface of lipid membranes can be neglected, assuming that head groups of the anionic phospholipids are fully charged.

## 4. Registration of the Boundary Potential of Planar Membranes (IFC Method)

**Figure 3:**
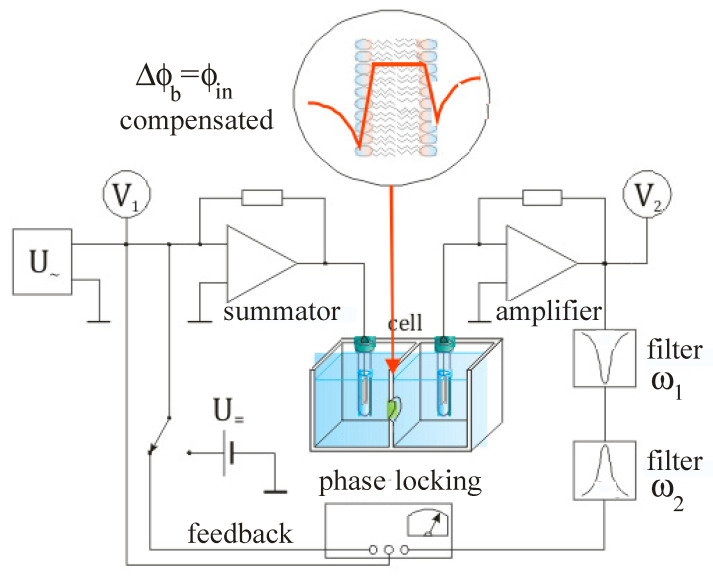
Scheme for measuring the difference of the boundary potentials of planar BLM using the amplitude and phase of the second harmonic of the capacitive current. In the inset, the red line shows the distribution of the electric potential in the membrane under conditions of compensation of the intramembrane field *φ_in_* [1,35].

A special point in the analysis of electrokinetic data is the choice of the parameter *δ* = 0.2 nm for the distance between the surface and the shear plane. Authors of [24,33] assume this parameter in the quantitative description of the EDL in the presence of monovalent and divalent cations, arguing for this choice by good agreement between the electrokinetic data and data for voltage-dependent fluorescent probes or conductivity of planar bilayer membranes (BLMs). In our experiments, we used a new technique developed for monitoring the boundary potentials of planar BLM using the method of intramembrane field compensation (IFC) [34]. A typical setup developed for this procedure is shown in Figure 3.

Measurements of boundary potentials of lipid membranes assume that planar BLM formed on a hole of about 1 mm in the cell septum. It can be performed by two ways suggested in the literature: from lipids solved in some organic solvent (typically, decane) [36] or by bringing together two lipid monolayers across the hole in a septum [13]. As was discovered earlier in [7], the electrical capacitance of these membranes depends quadratically on the magnitude of the external field. Later, it was quantitatively related to membrane electrostriction phenomenon [37,38].

The phenomenon of electrostriction is observed when the thickness of the lipid bilayer and its electrical capacitance change in the presence of a difference in boundary potentials ∆*φ* = *φ_b_*_1_ − *φ_b_*_2_ across the membrane. This difference arises due to the asymmetric charge distribution and dipole moments on both sides of the membrane. As is shown in [39], the dependence of the capacitance on the external voltage leads to the appearance of higher harmonics of the capacitive current, of which it is the amplitude of the second harmonic that is directly proportional to the constant component of the external voltage, *U*_0_. In our studies, we have been able to use this fact to construct a convenient electrical circuit for recording the boundary potential difference [35] and have developed a technique to analyze electrostatic fields at the interface of lipid membranes [1]. Using an external voltage source, *E*, the difference ∆*φ* can be compensated with good accuracy by an electronic circuit, an approximate view of which is shown in Figure 3. Components of this circuit can be implemented by analytical instruments and software.

The effect of an electric field on the capacitance of the lipid membrane is well described by simple relationships that follow from the elastic capacitor model. In the first approximation, the electric capacitance of such a capacitor has a quadratic dependence on the potential difference between its planes [35],

(6) $$C=C_{0}\left[1+\alpha {\left(U-{\Delta \varphi }_{b}\right)}^{2}\right]$$

where *α* is the coefficient of its elasticity. If the external field consists of the variable *V* and constant *U*_0_ components, *U* = *U*_0_ + *V*, the electric current through the capacitor contains high harmonics, the first three of which have a different relationship with the AC and DC components of the electric field:

(7) $$I=-V\omega C_{0}\left(1+3\alpha U_{0}^{2}+\frac{3}{4}\alpha V^{2}\right)\sin\left(\omega t\right)-3\alpha C_{0}\omega U_{0}V^{2}\sin\left(2\omega t\right)-\frac{3}{4}\alpha C_{0}\omega V^{3}\sin\left(3\omega t\right)$$

Note, the amplitude of the first harmonic is proportional to the electric capacitance of the membrane, the third depends only on the variable component of the electric field, which makes it possible to estimate the elasticity constant of the capacitor, but the second harmonic depends on the *U*_0_ which may compensate the boundary potential difference between two BLM sides. The electrical circuit assumes the selection of the signal of the second harmonic of the capacitive current using its amplitude in the proper phase as negative feedback. As a result, the compensation state corresponds to a zero magnitude of the second harmonic signal and to the equivalence ∆*φ_b_* = *φ_in_* [1,35]. Therefore, the IFC method allows for the monitoring of the kinetics of adsorption of any substances at one membrane side by keeping the other one as a reference.

The principal limitation of the IFC method is the increased permeability of the membrane for ions, which is possible for various reasons. However, inorganic ions and many biologically important molecules do not penetrate the membrane, creating an asymmetric distribution of boundary potentials. The kinetics of their adsorption at the BLM surface can easily be monitored even in the case of continuous change in the solution by perfusion. Thus, in particular, it is possible to experimentally establish the degree of reversibility of the process of the binding of ions and molecules to the membrane surface that is especially important in studying the adsorption of large molecules of synthetic and natural peptides [40].

**Figure 4:**
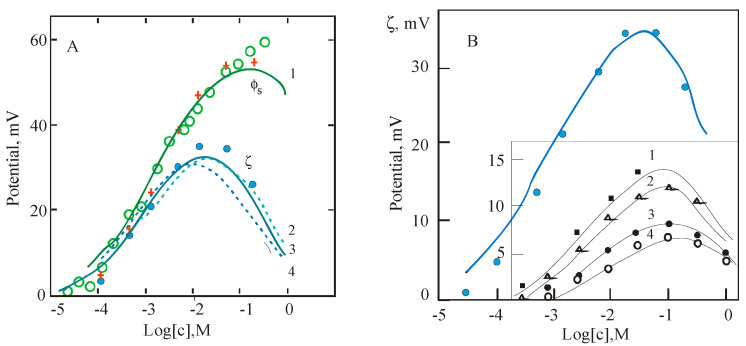
(**A**). The surface potential (green points and line) and ζ-potential (blue points and lines) measured by increasing the concentration of BeSO_4_ in the background electrolyte (10 mM KCl) at one side of planar BLM and in the liposome suspension made from neutral PC lipids, respectively. Theoretical curves present GCS model prediction with a shear plane at a distance of 0.2 nm (solid curves and red crosses) or for varied production of this parameter and surface charge density (dotted line) as described in the text below. (**B**). Experimental data for Be^2+^ are compared with other divalent cations reproduced from publications [34,41]. Details are in the text.

The typical procedure of measurements using the electrokinetic method and detection of boundary potentials of planar BLM consists of a successive increase in the concentration of the studied ion in the experimental cell. Figure 4 shows the results of such measurements for the adsorption of some divalent cations at the surface of electrically neutral phosphatidylcholine (PC) membranes. The adsorption of multivalent cations, measured by electrokinetic methods for zwitterionic and therefore neutral lipids, is well described by the GCS model with a simple expression [2,27]:

(8) $$\frac{\sigma }{\sigma_{max}}=\frac{z_{2}K_{2}c_{2}\left(0\right)}{1+K_{2}c_{2}\left(0\right)}$$

Here *c*_2_ is the concentration of cations of the valence *z*_2_ adjacent to the membrane surface and the binding constant *K*_2_ is related to the cation adsorption equilibrium. The surface charge density is increased with cation adsorption up to its maximal value, which is determined by the density of binding sites associated with each lipid head group. Formally, it corresponds to the density of lipids in the bilayer.

Electrokinetic data for the adsorption of beryllium cations at the surface of liposomes from neutral PC are presented in Figure 4A by blue points and the theoretical blue solid curve. Data of the IFC method for boundary potential at one side of the BLM of the same lipid are shown by empty green points. The red crosses correspond to the surface potential calculated from the electrokinetic data by Equation (3) a in framework of the GCS model, taking into account the position of the shear plane at the distance *δ* = 0.2 nm. Good agreement between electrokinetic and IFC data confirms the choice of GCS parameters.

Note, two more dotted theoretical curves in Figure 4A were found in the framework of the GCS model and turn out to be close to the experimental ones even with a significant variation in the production of the charge density and the distance from the surface to the hydrodynamic shear plane. The theoretical curves were plotted at the values of *σ_max_* (C/m^2^) and constant *K* (M^−1^), respectively, 0.2 and 400 for curves 1 and 2. For the other curves, the values of these parameters were taken equal to 2 and 40 (curve 3), 0.02 and 4000 (curve 4). The maximum density of phospholipids packed into a bilayer, when evaluated for the case of each molecule carrying a single charge, approximately corresponds to *σ_max_* = 0.2 C/m^2^. Just this value, as well as the binding constant of Be^2+^ cations, equal to = 400 M^−1^, and the position of the shear plane, are well suitable as parameters for the GCS model. This model gives a reasonable magnitude of the surface potential and cation binding constants from the results of the electrokinetic measurements.

As follows from the data in Figure 4B, experiments with other divalent cations show non-monotonic variation of ζ-potential in approximately the same concentration range. Based on the calculations performed using the isotherm (8) and comparing the electrokinetic data with measurements of the boundary potential on planar BLMs (Figure 4A), we may conclude that the ζ-potential decreases in the same way for all divalent cations according to their contribution to the ionic strength of the electrolyte. The screening of the surface charge is responsible for this effect. Our electrokinetic data for Be^2+^ are combined in Figure 4B (inset) with data from the literature for a number of divalent cations given in the review [41]. A series of curves in this figure reflects the adsorption at the surface of phosphatidylcholine liposomes of divalent cations in BeSO_4_ solutions (upper curve, our data) and CaCl_2_, MgCl_2_, SrCl_2_, and BaCl_2_ solutions for curves 1–4, respectively. In our numerical estimates, the magnitude of the maximum of these curves on the scale of potentials only indirectly reflects the difference in the cation binding constants. To get a real constant, one needs to make a correct numerical analysis with the value *δ* = 0.2 nm. Unfortunately, a fairly simple equation for estimating these constants, proposed in the above-cited paper [41], does not take into account the position of the shear plane.

**Figure 5:**
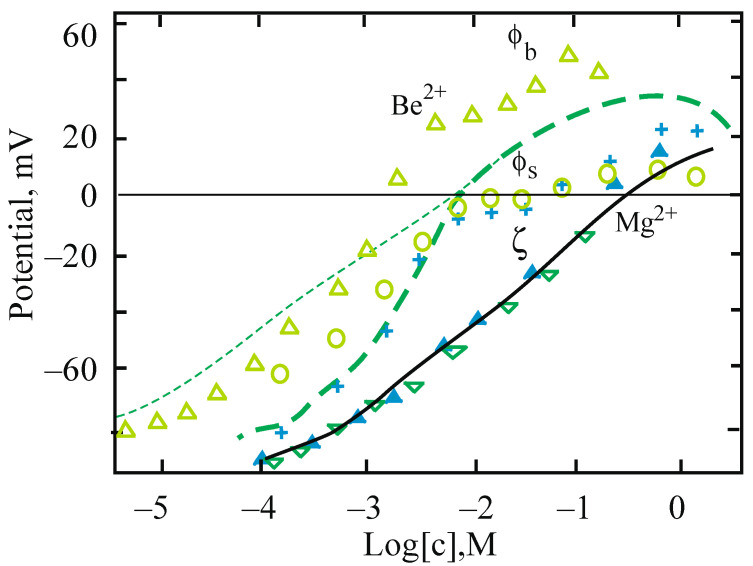
Boundary potential of the planar BLM (light and dark green triangles) and ζ-potential of liposomes from phosphatidylserine (blue crosses and triangles), measured at different concentrations of divalent cations Mg^2+^ and Be^2+^, respectively, in a background electrolyte (100 mM KCl, 20 mM imidazole, pH = 6.4). The values of the surface potential were calculated for the adsorption of Be^2+^ (light green circles) and Mg^2+^ (dark blue triangles) within the framework of the GCS model, taking into account the hydrodynamic shear plane at a distance of *δ* = 0.2 nm. The boundary potentials are registered at one side of the planar BLM by the IFC method and then shifted to a level of −85 mV, which corresponds to the surface potential found in the electrokinetic measurements. (According to [42,43,44]).

Electrokinetic measurements in a liposome suspension and IFC with planar BLM, have been applied for intensive studies of the adsorption of multivalent cations at the surface of negatively charged phosphatidylserine membranes. Experimental data of both methods are presented in Figure 5. In the case of Mg^2+^ cations with a small attraction to the lipid surface, they demonstrate a good agreement with the parameters of the GCS model and take into consideration the position of the hydrodynamic shear for ζ-potential analysis. The isotherm for competitive adsorption of cation with the arbitrary valence *z* at the surface of membranes from anionic lipids has the following form [2,27]:

(9) $$\frac{\sigma }{\sigma_{max}}=\frac{\left(z_{2}-1\right)K_{2}c_{2}\left(0\right)-1}{1+K_{1}c_{1}\left(0\right)+K_{2}c_{2}\left(0\right)}.$$

Here, indices 1 and 2 refer to the monovalent cation of the background solution and the multivalent cation of the valence *z* present in it. In particular, from this expression and the data presented in Figure 5, it can clearly be seen that in the case of Mg^2+^, the binding constant can be found directly from the position of the zero charge point in the concentration scale, where *K*_2_ *C*_2_(0) = 1. It corresponds to the negligible electrophoretic mobility and a surface charge density equal to zero. The same result has been obtained with other biologically important cations Ca^2+^ and Ni^2+^. Note, this conclusion does not depend on the position of the shear plane, which must be taken into account in all alternative cases. Good agreement between the two methods is observed for these cations because the dipole component of the boundary potential remains unchanged. At the same time, this component is the most sensitive to changes in the structure of the lipid bilayer, which occur, for example, when the phase state of the lipids changes.

The equilibrium constants of cation binding and other parameters of the GCS model were obtained by approximating the experimental data shown in Figure 5 using isotherm (9) and have the following values: *K*_2_ = 1000 M^−1^ for Be^2+^ and *K*_1_ = 0.6 M^−1^ for potassium. The value of the maximum charge density *σ_max_* = 0.2 C m^−2^ is taken for all curves under the assumption that each lipid molecule is a single charged center of the competitive binding of cations. The thin dotted line in Figure 5 corresponds to the original GCS model with no depletion effect. In contrast, theoretical curves for the adsorption of Be^2+^ cations, the condition of material balance (see (10) below) and depletion of solutions have been used due to the high efficiency of adsorption of this cation with a conditional lipid concentration *c_lip_* = 0 for the curve with the short-dotted line and *c_lip_* = 1 mM for the curve with the long-dotted line. This factor becomes very essential for ions and substances of extremely high affinity to the lipid surface. This will be discussed in more detail for the case of the adsorption of lanthanides.

A significant change in the dipole component of the boundary potential has been observed for some multivalent cations that are capable of initiating phase transitions in the lipid bilayer, when the membrane contains anionic phospholipids, for example, PS molecules [43]. In this case, we are interested in the fact that the adsorption of this cation significantly affects the course of many biochemical intracellular processes and, in particular, the fact that PS molecules play an important signaling role [45]. The change in the dipole component of the boundary potential in the presence of this cation clearly indicates a change in the packing of this lipid in the membrane. A series of experiments with this cation in a suspension of erythrocytes show that its adsorption disrupts the natural process of the interaction of these cells with macrophages responsible for removing old cells from the body [44]. There is a reason to believe that this is the cause of the initial stages of a serious disease—berylliosis.

It should be noted that the adsorption of beryllium cations leads to a change in the electric field at the boundaries of neutral lipids as well. The corresponding measurements were carried out by us both with liposomes and planar BLMs from DPPC at temperatures below and above the temperature of the phase transition of this lipid between the LE and LC states, and calorimetric measurements revealed signs of lipid condensation [43]. At the same time, the electrokinetic measurements in both cases have been well described within the framework of the GCS model, if data on the temperature dependence of the dielectric characteristics and the viscosity of the aqueous medium are used. Measurements with BLM at low temperatures did not take place due to the poor stability of lipid membranes in the condensed state. However, it turned out that when the membranes are cooled, the direction of changes in the boundary and ζ potentials have an opposite sign. The hypothesis about the effect of cations on the dipole component of the boundary potential has been tested and confirmed by calculations using molecular dynamics (MD) methods [31,46].

**Figure 6:**
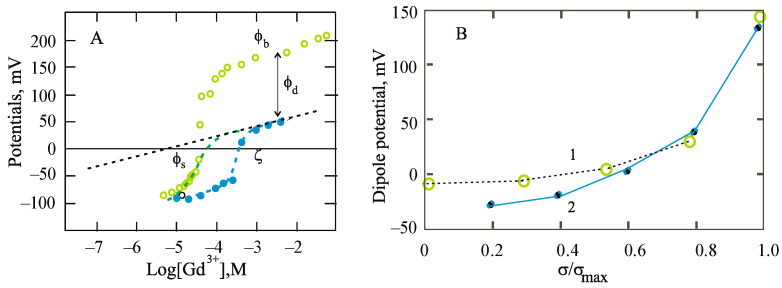
(**A**). Measurements carried out with a successive increase in the concentration of Gd^3+^ cations at one side of the planar BLM (light green points) and in the suspension of liposomes (dark blue points), respectively. The boundary potential is shifted to a level of −114 mV, which corresponds to the surface potential found in electrokinetic measurements of the ζ-potential of phosphatidylserine membranes. The dotted lines are the theoretical curves followed from the classic version of the GCS model (straight line) or corrected by the condition of the material balances with parameter *c_lip_* = 0 or 1 mM for planar BLM and liposome suspension, respectively. (**B**). The difference between the boundary and surface potentials corresponds to the dipole component at different ratios of *σ/σ_max_*_,_ which follows from two set of measurements: (i) with a variated portion of charged lipid PS in the mixture with neutral PC lipid (green points), and (ii) a background solution of different pH (blue points). According to [32,42]. Explanations are in the text.

Another important consequence of structural changes in membranes detected by electrochemical methods monitoring changes in the dipole component of the interfacial potential is clearly shown in Figure 6.

Interest in the adsorption of lanthanides on lipid membranes is caused by the fact that they are able to block mechanosensitive channels presented in the cell membranes of various organisms [47,48,49] and, in particular, in the membranes of *E. coli* [50]. Many researchers assumed a direct influence of the interaction of lanthanides with peptide subunits, preventing the normal functioning of proteins [51,52]. The phospholipids surrounding them were considered to be only the hydrophobic environment necessary for the fixation of proteins in the membrane. As it turned out in our experiments, this environment is able to directly regulate the conformational mobility of membrane proteins. This conclusion has been proved by the comparison of electrostatic and calorimetry data, which is directly related to dipole potentials with a condensed state of lipids [42,53].

The results in Figure 6 show the boundary and ζ-potential measured in the presence of Gd^3+^ cations in the background solution (50 mM KCl, pH about 7.0). At high concentrations of gadolinium at membranes with a maximum content of the charged form of PS molecules, the difference between these potentials reaches 150 mV. This difference is naturally attributed to the dipole component of the boundary potential. Note that the point of intersection of the abscissa axis differs by more than an order of magnitude, and the slope of the experimental curves near this point significantly exceeds that expected in the framework of the GCS model for trivalent electrolytes (about 20 mV per decade, [2]). This is due to the extremely high efficiency of the binding of this cation, at which the solution of this cation becomes depleted in the same way as in the previous case of the adsorption of Be^2+^. For a quantitative description of this effect, it is necessary to supplement the equations of the GCS model with the material balance condition:

(10) $$C_{tot}=C_{bulk}+C_{dif}+C_{ads}$$

(11) $$\frac{C_{dif}}{C_{bulk}}=\Omega \frac{A}{V}$$

Here Ω is the specific value of the excess concentration of ions per unit area, which can be found experimentally with significant changes in the dipole component of the boundary potential, taking into account the Boltzmann relation and the potential profile (3) in the diffuse part of the electrical double layer.

Theoretical curves shown in Figure 6 are constructed using these relations and the equations of the GCS model with the following values of the parameters [53]: *K*_1_ = 4 M^−1^, *K*_2_ = 5 × 10^4^ M^−1^. The material balance condition takes into account two values of the parameter c_lip_, which characterizes the size of the area available for cation adsorption. When describing experiments with planar BLMs about 1 mm^2^ in size, this value is taken to be equal to 0, and in electrokinetic measurements in a suspension of liposomes, it is equal to 1 mM, which approximately corresponds to the lipid content in the experimental cell. If all lipid molecules, the effective concentration of which in the cell is equal to *c_lip_*, and the area occupied by each molecule, *A_lip_*, in the monolayer, are available for ions, then the numerical value of the *A*/*V* ratio (m^−1^) is determined by the area of the monolayer built from all phospholipid molecules: *A/V* = *N_A_* × *A_lip_* × *c_lip_* 1000 = 3.6 × 10^8^ *c_lip_*, where *N_A_* is Avogadro’s number.

**Figure 7:**
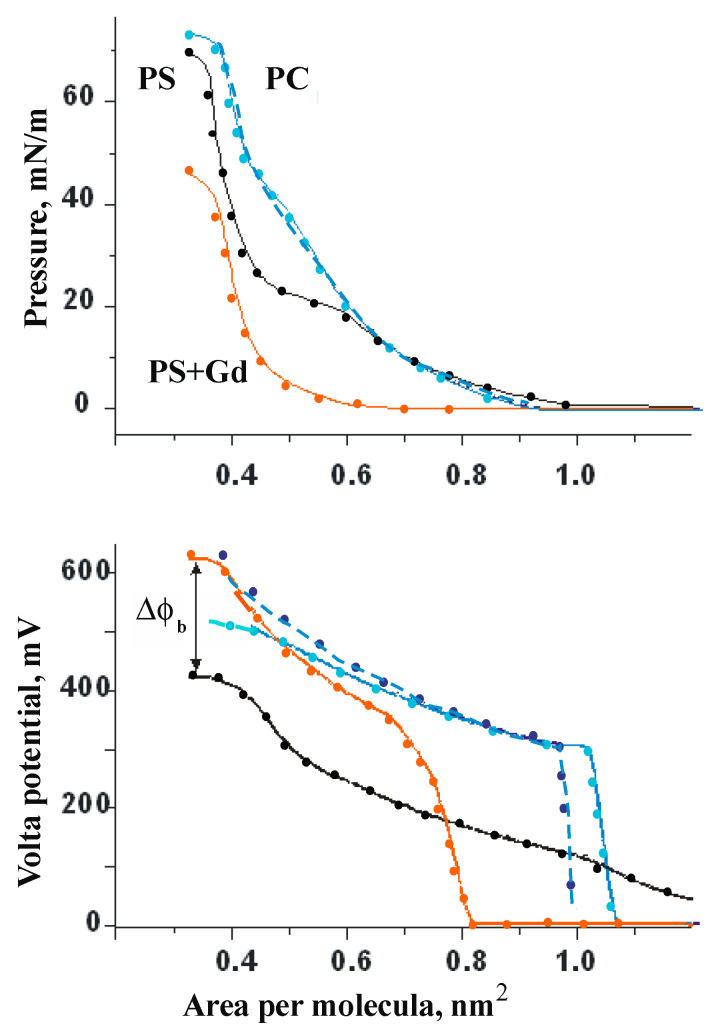
Compression and Volta potential diagrams of the lipid monolayers of DMPS and PC measured in a background electrolyte 0.01 M KCl (black and blue curves), and in the presence of 10^−5^ M GdCl_3_ (red and dark blue). Experimental curves are marked by solid points. According to [53].

The experimental data shown in Figure 6B demonstrate the difference between the boundary and surface potentials in experiments in which the surface charge density was varied by changing the pH in a suspension of liposomes from PS or by changing the content of this lipid in a mixture with PC [32,54]. As it turned out, when gadolinium is adsorbed on neutral phospholipids they acquire a positive charge, which is revealed in electrokinetic measurements, but there is no difference in the change in the boundary and surface potentials. Therefore, in the analysis of experiments with mixtures of lipids, it turned out to be sufficient to take into account the binding of the cation to the neutral regions of the membrane according to Equation (8), with a constant equal to 10^3^ M^−1^. [53]. These lipids were not involved in the experiments with the varying of the pH of the background solution. As follows from this series of measurements, the change in the boundary potential contains two components: as the concentration of the trivalent gadolinium cation increases, the surface potential of the charge density of the negatively charged surface is shifted to the positive direction and reaches the point of zero charge at micromolar concentrations of the cation. Judging by the measurements of the boundary potential, it is after the neutralization of the surface near a concentration of 10^−5^ M Gd^3+^ that it differs significantly from the surface potential, exceeding it by about 150 mV. The changes in the dipole component found in these two series of experiments practically coincide. Thus, the important role of the ionic form of phosphatidylserine molecules in these effects, which can cause structural changes in the lipid bilayer, has been proved. This fact is especially clearly demonstrated in experiments with lipid monolayers [53] (Figure 7).

## 5. Diagrams of the Compression and Volta Potential of Lipid Monolayers

Several experimental curves measured on monolayers of PS dimyristoyl derivatives (DMPS) are shown in Figure 7. In these experiments, along with the measurement of monolayer compression diagrams, changes in the Volta potential have been recorded, which in this case reflect changes in the boundary potential of lipid membranes.

The upper diagram clearly shows that the presence of gadolinium cations in the subphase does not affect the compressibility of a monolayer of neutral lipid PC, but makes it much less compressible and practically eliminates that part of the compression diagrams of the charged lipid DMPS, which usually corresponds to the liquid expanded (LE) state of the lipids. This fact, according to the data of the Volta potential (lower diagram), is accompanied by a significant increase in the potential by about 150 mV, as is the increment of the dipole component of the boundary potential in experiments with planar BLMs in Figure 6. The most natural explanation for these facts seems to be the lateral condensation of lipids initiated by the adsorption of gadolinium cations up to the complete neutralization of the surface. Independent measurements with liposomes from DMPS in the presence of gadolinium cations have been carried out by us by titration calorimetry and clearly showed the effect of this cation on the change in the phase state of the lipid from LE to the condensed (LC) state at cation concentrations close to the point of zero charge [53]. This not only confirmed the conclusions from previous measurements, but also provided the basis for a hypothesis about the most probable mechanism of the influence of this cation on the functioning of mechanosensitive channels. It is natural to assume that the condensation of anionic lipids in the environment of the protein, caused by this cation, prevents the normal conformational mobility of its subunits that is necessary to increase the size of the transmembrane channel. One may find the most recent review of hypotheses related to this subject in [55].

**Figure 8:**
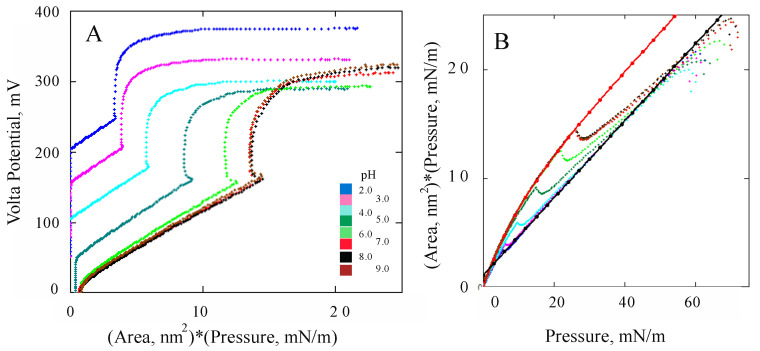
(**A**). A series of experimental curves obtained by measuring the Volta potential and compression diagrams of DMPS monolayers at various pH values in a 10 mM KCl background solution. (**B**) The same data presented in modified scales (see text below). Asymptotes are marked by red and black solid points. According to [53,57].

Area per lipid and lateral pressure at the point of DMPS phase transition varied with the state of ionization of the lipid. This effect was observed in our earlier experiments with DMPS monolayers at varied pH levels and ionic strengths of the aqueous subphase [53]. The initial experimental curves in Figure 8 are presented at different scales. The shift of the Volta potential with pH reflects a variation in lipid ionization with *pK* = 3.0 and corresponds to proper changes in the negative surface charge, in full accordance with the data of previous measurements [32]. Figure 8A shows the changes in the Volta potential as a function of the product of the area per molecule and the lateral pressure, which has the energy dimension. The experimental data are presented in Figure 8A in scales that make it possible to clearly demonstrate the potential jump during the phase transition from the LE to the condensed LC state. When the pH and the density of the negative charge on the surface increase, the transition point shifts towards greater work being necessary to compress the monolayer. This seems quite natural, since an excess charge among the lipid molecules leads to their electrostatic repulsion and more energy is required to reach the condensed state. Another feature noticeable in these curves indicates a linear relationship between Volta potential changes and the energy in the region where the phospholipid molecules remain in the liquid-crystalline LE state. This fact is also observed for monolayers of other phospholipids [56]. The same experimental curves are presented in Figure 8B in other coordinates, where the asymptotic curves become visible and the monolayer compression work as a function of the lateral pressure is manifested. These asymptotes are quantitatively described this work (a production of area and pressure) in different forms: *AP* = *A*_0_ × *P* + *A*_1_ × *α*[1 − $exp^{-P/\alpha }$]—for the upper asymptote and linear dependence *AP* = 10^9^ + *A*_0_ × *P*—for lower asymptote. Here, the area per molecule in the initial state is fixed at *A*_0_ = 3.55 nm^2^, the additional area in the rarefied state at *A*_1_ = 5.7 nm^2^, and the compression elasticity constant expressed in units of pressure *α* = mN/m.

It should be noted that theoretical description of compression diagrams and their transformation at the phase transition in the lipid monolayer has attracted the attention of many researchers. A sufficiently substantiated model of the phase transition was proposed, for example, by the authors of [58,59]. The authors described phase fixed transition as the equilibrium of free molecules of lipid redistributed between the LE region and LC clusters. Unfortunately, this model has been applied only to neutral lipid monolayers, where electrostatic effects are minimal and the value of the Volta potential has not been controlled in the experiment. But the authors suggest a fruitful idea about ion and water effects on the interface tension [60,61]. According to our data, the work applied to compress the monolayer of anionic lipids is partly spent on charging them. This fact can be demonstrated by a simple analysis of the experimental curves, which reveals a direct relationship between the increment of Volta potential, ∆*φ* and the decreased area during the monolayer compression, Δ*A*. This analysis is made in the work [56] for a series of curves measured on DMPS monolayers in a wide range of background electrolyte concentrations. A linear relationship between these quantities is established: ∆*φ*/∆*P* = −Q (∆*A*/∆*P*). The proportionality coefficient *Q* has a value of approximately 0.5 V/nm^2^ in all sections of the experimental curves measured at KCl concentration in aqueous solutions that varied from 10^−3^ to 1 M.

Recently, the physical reasons for Volta potential behavior in different parts of the monolayer compression curve have been revealed in [62], in which the interface of DMPS monolayers at several points of compression was studied by the methods of the small-angle scattering of X-rays and molecular dynamics. The charge distribution over lipid–water interface was evaluated from the intensity of X-rays in a direction normal to the monolayer surface. The same quantity was calculated from the molecular structure, visualized by MD methods. Good agreement between the results of both methods clearly proves the molecular structure and thus makes it possible to reveal fine details of the difference between the LE and LC states of lipids and to recover the important physical events at the phase transition. It becomes clear that in the region of the LE state, water molecules are gradually “squeezed out” from the hydration shell of the phospholipid, and thereby the total dipole moment in the polar region of the interface changes. During a phase transition, this process is sharply disturbed, lipid molecules approach each other so close that a further decrease in the distance between them is reflected mainly in the orientation of the dipole moment of methyl groups at the end of hydrocarbon chains. The dipole effects associated with the molecular structure of lipid chains are studied in more detail using the technique of lipid monolayers in [63,64]. The presence of an excess charge in polar groups, of course, requires a greater lateral pressure for such an approach by the molecules. This study greatly improves the understanding the relationship between electrostatic phenomena and the structure of the lipid–water interface.

## 6. Ionized Amphiphilic Molecules at the Interface of Membranes

The above interpretation is helpful in constructing a model and a quantitative description of the effect of the incorporation of molecules of the pharmacological drug chlorpromazine (CPZ) into the lipid bilayer and monolayer. This substance is of special interest in pharmacology because it is partially hydrophobic and therefore its incorporation into cell membranes dilutes the lipid matrix and makes it softer in the lateral direction [65,66]. At the same time, it is positively charged and electrostatic phenomena induced by its adsorption at lipid membranes opens up more complicated features than simple inorganic ions.

Similar to many membrane-active molecules, molecules of CPZ that carry a positive charge under normal conditions also display hydrophobic properties. An empirical analysis of the diagrams of compression and the Volta potential in experiments with these molecules is presented in [57] based on the assumption of a linear relationship between the relative increase in the area *A* and the lateral pressure in the monolayer [3,67]. The compression of a monolayer in the pressure range, which corresponds to the “liquid” LE region in the pressure-area diagrams, can be represented as a decrease in the excess area of molecules proportional to the increase in pressure in the monolayer, *P*:

(12) $$\frac{dA}{A-A_{0}}=-\frac{dP}{K_{P}},$$

where *K_P_* is the isothermal lateral compression modulus, *A*_0_ is the minimum incompressible area of a lipid molecule. In the general case, integration (12) with the boundary condition in the high-pressure limit *A* → *A*_0_ leads to the exponential pressure dependence of the area.

(13) $$A=A_{0}+A_{e}e^{\left(-\frac{P}{K_{P}}\right)}$$

Parameters in (13) are the minimum area of the molecule, *A*_0_, which does not depend on the applied pressure as the rigid “core” is surrounded by a soft “shell” with an area equal to *A_e_* at zero pressure. Using the fact of the linear relationship between the Volta potential and the work on compression of the monolayer, one can compare the change in the potential with the presence of CPZ molecules in the subphase in the region where the behavior of lipids corresponds to the LE state. The charging of the interfacial boundary of the monolayer on the electrolyte surface reflects the work, *W*, spent on its compression:

(14) $$W=A_{0}P+A_{e}Pe^{\left(-\frac{P}{K_{P}}\right)}$$

To quantitatively describe the process of interfacial boundary charging, the work on compressing the monolayer, *W*, should first of all be supplemented by an estimate of the energy expended for embedding charged molecules into it. The concentration of charged chlorpromazine molecules near the surface, *C_s_*, is related to their concentration in the subphase, *C*_0_, by the Boltzmann relation:

(15) $$C_{s}=C_{0}e^{\left(\frac{e\varphi }{kT}\right)}$$

The surface density of the molecules built into the bilayer is proportional to their near-surface concentration *C_L_* = *K_d_* × *C_s_*, where the constant, according to electrokinetic measurements, is equal to *K_d_* = 0.5 M^−1^. The incorporation of new molecules into a monolayer requires energy and is accompanied by an increase in lateral pressure. This additional energy can be taken into account by an expression similar to the Boltzmann relation (16) provided that the concentration of embedded CPZ molecules is related to the pressure in the monolayer by the expression:

(16) $$C_{L}=K_{Ld}e^{\left(\frac{-P}{K_{P}}\right)}$$

This expression is similar in form to the Boltzmann relation for the energy of a CPZ molecule in a monolayer, if the value of the constant *Kp* is expressed in units of *kT* and the characteristic area is entered as *A_ch_* = *kT/K_p_*. Comparison of relations (15) and (16) leads to a linear dependence of the diffuse component of the potential, *φ*, on the pressure in the monolayer:

(17) $$\frac{e\phi }{kT}=-\frac{P}{K_{P}}+const,$$

where const = ln(*K_Ld_/K_d_C*_0_). Hence, it follows that, with increasing pressure, the potential increment caused by the incorporation of positively charged molecules into the monolayer should decrease, and the Volta potential should shift in the negative direction, i.e., approach its value for the initial monolayer of negatively charged DMPS molecules. Ultimately, the work of compressing the monolayer, W, is described by the expression:

(18) $$W=AP+\frac{kT}{e}\left[-\frac{P}{K_{P}}+const\right]$$

**Figure 9:**
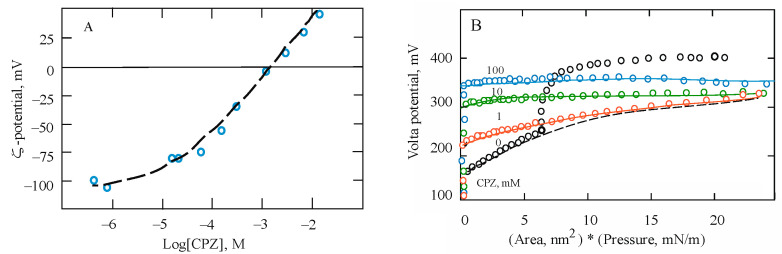
(**A**). ζ-potential of liposomes from PS, measured at different concentrations of CPZ. The theoretical curve was plotted according to the GCS model and the Langmuir isotherm (11) under the condition of adsorption of CPZ with one positive charge and a binding constant of 0.5 M^−1^. (**B**). Results of the measurements of compression and the Volta potential diagrams of DMPS monolayers in the presence of CPZ molecules in a background electrolyte of 10 mM KCl. The theoretical curves are constructed according to the model whose equations and parameter values are given in the text. According to [57].

Assuming, as before, that the charging of the interface is proportional to the work expended on compressing the monolayer, the experimental dependences in Figure 9B were quantitatively described by the expression (with the exception of a pure DMPS curve in the solid LC state):

(19) $$\Delta E=N\left[A_{0}P+A_{e}Pe^{\left(-\frac{P}{K_{P}}\right)}\right]+D\left[-\frac{P}{K_{P}}+const\right]+\varphi_{0}$$

where the numerical values of the parameters *N*, *D*, and const given in Table 1, were found during data approximation. We also note that the value of the potential at the beginning of each experiment occurs largely for random reasons, while the parameters *N* and *D* determine, respectively, the contribution to the Volta potential of the elastic characteristics of the monolayer and the process of “squeezing” charged CPZ molecules out of it.

Judging from the experimental data shown in the figure and the model parameters in Table 1, the incorporation of positively charged molecules into the monolayer shifts the values of the Volta potential in the negative direction, bringing it closer to the potential of the initial DMPS monolayer. In this case, the charging of the interface is proportional to the work spent on compressing the monolayer. Increasing the CPZ concentration shifts the curves by *φ*_0_, but this value at the beginning of each experiment is determined by random causes, and the ratio of other parameters is more informative. Thus, the parameters *N* and *D* determine the contribution to the potential of the elastic characteristics of the monolayer and the process of extrusion of CPZ molecules from it. In this case, the cooperative process of phase transition of the monolayer to the condensed state occurs only after the removal of foreign molecules, which are not only CPZ molecules, but also the hydrated layer. Judging by our estimates, the contribution of CPZ molecules to the increase in the rigidity of the monolayer and the work of its compression remains the same at all concentrations (parameter *D*), and the fraction of the energy of the lateral interaction of lipid molecules (parameter *N*) decreases with the concentration of CPZ due to a decrease in the contact between phospholipid molecules. Of course, such an interpretation remains speculative and can only be confirmed by a detailed analysis of molecular dynamics data, which requires a separate study.

## 7. Molecular Structure of Interfaces

The application of the GCS model and the determination of parameters close to the experimental ones have to be correlated with the molecular structure of the bilayer visualized by MD methods. This is a very difficult task because of the very variable orientation of molecules along the interface with the aqueous medium. Another problem is to correlate the position of inorganic ions at the surface and in the diffuse part of the electrical double layer. In other words, to fix the internal and external Helmholtz planes in such way that the equilibrium of ions and their adsorption at the surface can be described by the parameters of the GCS model. A solution for this problem is suggested in [31,46] for the case of Be^2+^, Na^+^, and Cl^−^ distribution at the surface of a DPPC membrane. According to the molecular structure visualized by MD, the maximum distribution of cations and anions differs significantly. The position of this maximum may be associated with the internal Helmholtz plane inside the polar region of the bilayer at different depths for cations. The outer Helmholtz plane, as a formal membrane surface, was chosen at the maximum Cl^−^ distribution that does not present inside the membrane. The position of these maximums and the Helmholtz planes changed at the lipid phase transition. The above-described interpretation makes it possible to correlate the electric potential variations observed in the real experiments and to evaluate binding constants in good agreement with electrokinetic data and the GCS model. Later, MD analysis of lipid monolayers was combined with X-ray measurements in [62] and succeeded in the interpretation of the Volta potential and the elastic characteristics of lipid monolayers during their compression. Within the framework of a brief review, we cannot go into the details of the analysis of such structures, which are described in sufficient detail in the publications cited above [31,46]. It is important to note here that, judging by the MD data, adsorbed cations affect the orientation of the polar groups of the lipid and a number of free or oriented water molecules at the interfacial boundary. It was found that, when lipid molecules passed to the compact packing, the dipole component of the boundary potential decreased as the number of adsorbed cations increased and, accordingly, changes in the surface potential move in a positive direction. In particular, the jump in the Volta potential, which is recorded in experiments on compression of a DMPS monolayer in the phase transition region, can be attributed to the loss of several water molecules and, for this reason, a change in the orientation of the ionized hydrocarbon chains of the lipid.

**Figure 10:**
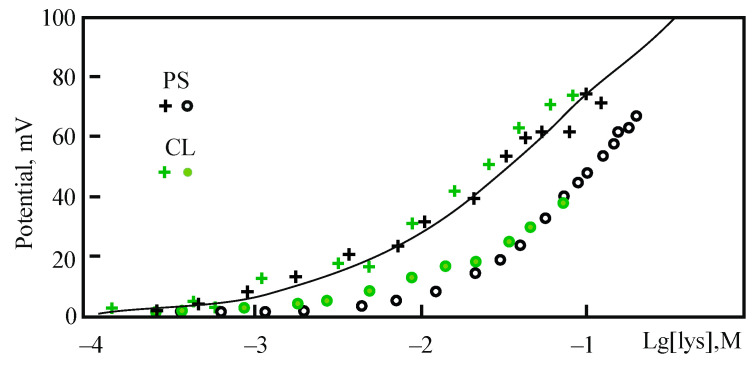
Lysine effect on the surface and boundary potential of membranes, composed in 10 mM KCl solutions from anionic lipids—phosphatidylserine (PS, blue symbols) and cardiolipin (CL, green symbols). Solid points are for the data of IFC methods applied to planar BLM, crosses are for the surface potential of liposomes made from the same lipids and evaluated on the base of the GCS model presented above. The theoretical curve corresponds to binding parameters 0.2 and 1.1 M^−1^ for potassium and lysine moieties (with a single positive charge), assuming their noncompetitive adsorption on the surface (adopted from [68]).

The application of the IFC technique makes it possible to measure the changes in the interfacial potential at the membrane boundaries that are also caused by large macromolecules significantly changing both the diffuse part of the electric double layer and the dipole component of the boundary potential. The first question regards the direct contact of charged units with the membrane surface, as follows from electrostatic measurements. The second one is how separate these effects are from the role of total chain length in these interactions. The best objects for this study are synthetic polymers with a linear structure based on one type of peptide such as polylysines of varied molecular mass. Figure 10 demonstrates results for lysine molecule adsorption at the negatively charged membranes.

Adsorption of lysine demonstrates unexpected facts. According to the data in Figure 10, the boundary potential remains unchanged up to the lysine concertation that corresponds to about 40 mV of surface potential registered in a liposome suspension. The only possible explanation is that the dipole potential is directed in the opposite direction to the surface. Note, this observation is different from that observed above with multivalent Gd^3+^ and Be^2+^. If the latter cases have been attributed to the lipid dehydration and condensation effect, lysine adsorption makes something different, possibly increasing the number of water molecules associated with lipid polar heads. This factor has been intensively discussed in the literature [10]. Moreover, lipid phosphates seem to be responsible for this effect because, for PS and CL, the effects are remarkably similar. This corresponds to the idea about dipole effects by the same authors published later [69].

It should also be noted that MD simulations show the role of H-bonds in the dipole effect caused by lysine adsorption [68]. This peptide reduces the number of H-bonds with water molecules around the carbonyl and phosphate groups of lipids. The latter, but not the carbonyl or carboxyle, is responsible for the changes in dipole potential found in the experiment. This is the reason for almost identical effects of the two types of lipids with different headgroups shown in Figure 10 [68]. Another interesting idea was recently suggested regarding dipoles in the polar headgroups of lipids. In the works [70,71], a new experimental system was used to detect changes in the bilayer capacitance after a special “learning” procedure by an external voltage applied to the membrane. Unexpectedly, these changes persist for a long time (tens of minutes). The authors interpreted this phenomenon as a model of “memory” at the molecular level. To explain the complex lipid mobility in similar systems, a phonon theory was developed in [72]. In the context of this review, it is interesting to mention that this “memory” effect depends on the ionic composition of the medium [73]. According to the authors, this is due to the influence of ions on H-bonds, which participate in lateral contacts between lipids. Direct electrostatic measurements seem to be most suitable to test this concept. Also, it would be desirable to confirm the experimental results using MD simulations.

Several experiments with polylysines of high molecular mass reveal two phase kinetics in boundary potential changes when added to one BLM side. The first one is fast and high *φ_b_* changes in the positive direction, similar to ζ-potential, but then the value of *φ_b_* slowly decreases to a stationary level. The concentration of polymer increased step by step and the total sum of positive changes directly correspond to surface potential in the electrokinetic measurements, which, by a sum of lower stationary levels became close to −40 mV of the “dipole” effect in the case of lysine (see [68] for other details). We may conclude that polylysine adsorption induces the same hydration and reorganization of hydrogen bonds under polymer domains.

The peculiarity of such objects is that when analyzing changes in the ζ-potential, it is no longer possible to apply the GCS model directly. This is caused, in particular, by the size of macromolecules, which occupy a significant part of the near-membrane space and violate the conditions of hydrodynamics, making it impossible to describe them within the framework of Smoluchowski’s model. This factor was discussed in serial works [74,75,76,77,78]. At present, this circumstance does not allow using simple methods to compare the changes initiated by them in the charge and potential distribution on the membrane surface and its vicinity with the number of adsorbed molecules, as was carried out for the adsorption of small molecules and ions. Moreover, their adsorption can be accompanied by a change in the conformation of the molecules themselves or by independently constructing such models in a highly simplified form for further calculations of the surface dipole reorientation, as a result of the monomer–dimer equilibrium [79]. An empirical approach assumes the charge migration between polymer chains and the lipid surface of varied composition [80]. Naturally, even in those cases when hydrodynamic phenomena can be ignored, the direct solution of the Poisson–Boltzmann equations is accompanied by the introduction of a huge number of parameters, the values of which are difficult to verify experimentally [81]. Fortunately, X-ray methods can be applied to study the interface of lipid monolayers and thus to estimate the charge distribution in a direction normal to the lipid monolayer and then to calculate the thickness of the polylysine layer [82]. A detailed analysis of these data makes it evident that polymer adsorption affects lipid hydration and removes water molecules from a polar region of the bilayer. Some theoretical approaches to these systems prefer to use the equations of the GCS model to describe electrokinetic data for the adsorption of positively charged macromolecules of polylisines of different lengths to the negatively charged. They lead to conclusions on the non-monotonic lateral distribution of these large polymers caused by their electrostatic repulsion compared to smaller ones [83,84]. A more detailed presentation of such studies leads us far from the main methodological theme of this review. Therefore, readers are recommended to refer to the literature cited above for a more accurate idea of the theoretical analysis in this area. Unfortunately, the direct calculation of hydrodynamics and field distribution using the Poisson–Boltzmann theory introduces many parameters that significantly complicate the EDL structure, but not all of them can be verified by experimental methods.

## 8. Conclusions

The above experimental facts and their quantitative analysis make it possible to formulate several important conclusions that follow from the observed changes in the electric field distribution at the membrane boundaries.

1. Just as with the initial control of new methods and the equipment developed for any electrostatic measurements, it is methodologically important to make a series of experiments with a systematically varied ratio of charged and neutral molecules at the surface. These types of measurements make it possible to verify that the general predictions of the classical GCS model are satisfied.
2. The change in the surface potential of membranes in a fairly good approximation can be estimated using the classical approach to the structure of the diffuse part of the electric double layer according to the Gouy–Chapman model supplied by the Langmuir isotherm. A significant limitation is postulating the position of the hydrodynamic shear plane far from the surface. In the specific case of a sufficiently smooth surface of liposomes, this parameter turns out to be close to 0.2 nm upon quantitative approximation of the data.
3. If the activity of ion binding is extremely high, and the surface available for ions is large, then their concentration in the volume of the solution decreases and it becomes necessary to supplement the model with the material balance condition. This includes ions distributed between the surface and the diffuse part of the electric double layer near it. However, the main provisions of the GCS model remain unchanged.
4. The dipole component of the boundary potential is not available for control by electrokinetic measurements and requires alternative methods that are sensitive to changes in the total boundary potential. These methods have been developed for planar BLMs and are in good agreement with measurements of the Volta potentials of Langmuir monolayers.
5. The dipole component of the boundary potential is particularly sensitive to changes in the hydration state of the membrane’s polar region. In the case of the liquid-crystalline LE state of lipids, the variation in the dipole potential reflects changes in the number, orientation, and hydrogen bonds realized by water molecules associated with the polar groups of lipids. When ionized groups of phospholipids are neutralized by ion adsorption, the effect of their lateral condensation and the possibility of transition to a condensed LC phase state appear. As a result, several water molecules are squeezed out from the membrane and the electric field distribution is changed because the dipole potential in the LC state is affected preferentially by the orientation of the hydrocarbon tails of the molecules. Approximately the same transition can be observed when binding large charged synthetic macromolecules and polypeptides. Note that in some cases the sign of the dipole effect appeared in the opposite direction to changes in the surface component of the boundary potential. This is presumably caused by the reconstruction of the lipid polar region by hydration.
6. Volta potential measurements during a compression of lipid monolayers reveal several important patterns. Changes in this potential are proportional to the energy spent on compressing the monolayer. Moreover, the increase in the potential is directly related to the change in the compression ratio of the monolayer in the entire region of the compression diagram, including the region of transformation from the liquid-crystal to the condensed state of the monolayer. If adsorbed molecules are able to incorporate into a lipid monolayer, their concentrations in the monolayer and near its surface are related by a relation similar to the Boltzmann relation, which describes the energy input of the incorporated molecules to the increment of two-dimensional lateral pressure.

## Figures and Tables

**Table 1:** Model parameters used in constructing the theoretical curves in Figure 9B.

CPZ, μM	*A*_0_, nm^2^	*A*_1_, nm^2^	*P*_0_, mN/m	*K_p_*, m/mN	*N*	*D*	Const	*φ*_0_, mV
0	5.16	3.51	0.55	10.1	55	0	0	−165
1	6.18	4.29	0.86	18.4	32	50	14	460
10	6.09	58.6	1.44	24.3	14	50	14	390
100	7.21	49.4	3.97	21.4	12	50	14	360

## Data Availability

All datasets are available on demand.
